# Supplementary material for: Adherence to a Cholesterol-Lowering Diet and the Risk of Pancreatic Cancer: A Case–Control Study
Source: Nutrients. 2024 Aug 1;16(15):2508. doi: 10.3390/nu16152508 (PMC11314520; doi:10.3390/nu16152508)
Supplement: Supplementary file 1 [file nutrients-16-02508-s001.zip › nutrients-3081096-supplementary.pdf]

nutrients MDPI

Table S1. Adjusted odds ratios (ORs) and corresponding 95% confidence intervals (CIs) for pancreatic cancer risk according to dietary component [a] and the cholesterol-lowering diet score, including patients with treated hypercholesterolemia. Italy, 1991-2008.

Dietary component		Score	Cases		Controls		OR (95% CI) [b]	OR (95% CI) [c]
Women	Men		n	(%)	n	(%)		
**Non-cellulosic polysaccharides soluble fibers (g/day)**, as a proxy of viscose fibers								
<6.9	<7.3	0	171	(52.5)	290	(44.5)	Ref.	Ref.
≥6.9	≥7.3	1	155	(47.5)	362	(55.5)	0.51 (0.36-0.72)	0.54 (0.38-0.77)
**Monounsaturated fatty acids (% of total energy intake)**								
<11.5	<12.0	0	59	(18.1)	175	(26.8)	Ref.	Ref.
≥11.5	≥12.0	1	267	(81.9)	477	(73.2)	1.38 (0.96-1.97)	1.39 (0.96-2.01)
**Legumes (servings/week)**								
<1	0	0	142	(43.6)	243	(37.3)	Ref.	Ref.
≥1	>0	1	184	(56.4)	409	(62.7)	0.79 (0.57-1.08)	0.84 (0.61-1.17)
**Saturated fatty acids (% of total energy intake)**								
≥11.2	≥9.7	0	172	(52.8)	313	(48.0)	Ref.	Ref.
<11.2	<9.7	1	154	(47.2)	339	(52.0)	0.78 (0.58-1.04)	0.82 (0.61-1.11)
**Seeds or corn oil (g/day per 2000 kcal),** as a proxy of phytosterol								
<3.1	<2.7	0	187	(57.4)	317	(48.6)	Ref.	Ref.
≥3.1	≥2.7	1	139	(42.6)	335	(51.4)	0.82 (0.60-1.12)	0.90 (0.65-1.24)
**Dietary cholesterol (mg/day)**								
≥303.7	≥235.1	0	171	(52.5)	272	(41.7)	Ref.	Ref.
<303.7	<235.1	1	155	(47.5)	380	(58.3)	0.67 (0.45-1.00)	0.70 (0.46-1.04)
**Glycemic index [d]**								
≥71.0	≥70.9	0	169	(51.8)	318	(48.8)	Ref.	Ref.
<71.0	<70.9	1	157	(48.2)	334	(51.2)	0.91 (0.67-1.22)	0.86 (0.63-1.17)
**Cholesterol-lowering diet score**								
0 to 2			59	(18.1)	61	(9.4)	Ref.	Ref.
3 to 4			172	(52.8)	356	(54.6)	0.49 (0.32-0.76)	0.50 (0.32-0.79)
5 to 7			95	(29.1)	235	(36.0)	0.44 (0.28-0.71)	0.50 (0.31-0.80)

[a] Cut-offs for each dietary component were selected by means a receiving operating characteristic (ROC) analysis separately for men and women. [b] Estimated by means of logistic regression models conditioned on sex and adjusted for study center, age, year of interview, education, and energy intake. [c] Estimated by means of logistic regression models conditioned on sex and adjusted for study center, age, year of interview, education, physical activity, diabetes mellitus, family history of pancreatic cancer, smoking habit, drinking habit, and energy intake. [d] White bread scale.
